# Impact of pneumococcal conjugate vaccine 13 introduction on severe lower respiratory tract infections associated with respiratory syncytial virus or influenza virus in hospitalized children in Ulaanbaatar, Mongolia

**DOI:** 10.1016/j.ijregi.2024.100357

**Published:** 2024-03-19

**Authors:** Lien Anh Ha Do, Naranzul Tsedenbal, Chimidregzen Khishigmunkh, Bazarkhuu Tserendulam, Lkhagvadorj Altanbumba, Dashtseren Luvsantseren, Munkhchuluun Ulziibayar, Bujinlkham Suuri, Dorj Narangerel, Bilegtsaikhan Tsolmon, Sodbayar Demberelsuren, Cattram Nguyen, Tuya Mungun, Claire von Mollendorf, Darmaa Badarch, Kim Mulholland

**Affiliations:** 1 New Vaccines Group, Murdoch Children's Research Institute, Melbourne, Australia; 2 Department of Paediatrics, The University of Melbourne, Melbourne, Australia; 3 National Center of Communicable Diseases, Ulaanbaatar, Mongolia; 4 Ministry of Health, National Center for Communicable Diseases, Ulaanbaatar, Mongolia; 5 Mongolian National University of Medical Sciences, Ulaanbaatar, Mongolia; 6 Expanded Programme on Immunization, World Health Organization, Ulaanbaatar, Mongolia; 7 London School of Hygiene and Tropical Medicine, London, United Kingdom

**Keywords:** Pneumococcal conjugate vaccine, Respiratory syncytial virus, Influenza virus, Lower respiratory tract infections

## Abstract

**Objectives:**

Limited data indicate a beneficial effect of pneumococcal conjugate vaccines (PCVs) on respiratory syncytial virus (RSV) and influenza infections in young children. We evaluated the impact of 13-valent PCV (PCV13) introduction on the incidence of severe lower respiratory tract infections (LRTIs) associated with RSV or influenza in hospitalized children.

**Methods:**

Our study was restricted to children aged <2 years with arterial oxygen saturation <93% and children with radiologically confirmed pneumonia nested in a pneumonia surveillance project in four districts of Ulaanbaatar city, Mongolia. We tested nasopharyngeal swabs collected on admission for RSV and influenza using quantitative reverse transcription-polymerase chain reaction. The impact of PCV13 on the incidence of LRTI outcomes associated with RSV or with influenza for the period April 2015-March 2020 was estimated. Incidence rate ratios comparing pre- and post-vaccine periods were estimated for each outcome for each district using negative binomial models and for all districts combined with a mixed-effects negative binomial model. Adjusted models accounted for seasonality. Sensitivity analyses were conducted to assess the robustness of our findings.

**Results:**

Among 5577 tested cases, the adjusted incidence rate ratios showed a trend toward a reduction in RSV-associated outcomes: all LRTIs (0.77, 95% confidence interval [CI] 0.44-1.36), severe LRTIs (0.88, 95% CI 0.48-1.62), very severe LRTIs (0.76, 95% CI 0.42-1.38), and radiologically confirmed pneumonia (0.66, 95% CI 0.32-1.38) but inconsistent trends in outcomes associated with influenza.

**Conclusions:**

No significant reductions were observed in any outcomes associated with RSV and influenza after PCV introduction.

## Highlights

• This is the first report on the impact of pneumococcal conjugate vaccine on respiratory syncytial virus (RSV)/influenza-associated lower respiratory tract infections in Mongolia.
• There was a trend toward reductions in RSV-associated lower respiratory tract infections end points during the post-pneumococcal conjugate vaccine period.
• There are epidemiological risk factors associated with severe RSV infections in Mongolia.

## Introduction

Worldwide, respiratory syncytial virus (RSV) is the leading cause of acute respiratory infections in pediatric populations [1]. Influenza is less frequent in young children; however, it is also recognized as one of the important viral pathogens of childhood pneumonia globally [2]. There are two RSV prevention products recently approved to prevent severe RSV disease in infants: a long-acting monoclonal antibody (Beyfortus^TM^ nirsevimab, Astra Zeneca and Sanofi) for neonates and infants entering their first RSV season [3,4] and a single dose of a maternal vaccine (Abrysvo^TM^, Pfizer) [5] to be administered in the third trimester of pregnancy. However, both strategies would not be immediately accessible in low- and middle-income countries (LMICs). Influenza vaccination is generally not included in national childhood or maternal immunization programs in many LMICs [2], whereas in Mongolia, influenza vaccination has been introduced since 2014 [6]. RSV and influenza have synergistic effects with *Streptococcus pneumoniae (S.pneumoniae)* infection, observed in *in vitro* studies and animal models [7], [8], [9], [10], [11], [12]. *S. pneumoniae* infections, including pneumonia, meningitis and septicemia, are also an important cause of morbidity and mortality among children, especially in LMICs [13].

Since 2000, when the first pneumococcal conjugate vaccine (PCV) was licensed, the use of PCV in high-income countries has led to substantial reductions in pneumococcal disease and associated mortality. PCV has also shown an impact on LRTIs associated with RSV and influenza in some settings [14], [15], [16], [17]. A retrospective time series analysis of US hospitalization data from 1992/1993 to 2008/2009 reported a significant decline in RSV-coded hospitalizations in children aged <1 year (−18.0%), 4 years after the 7-valent PCV (PCV7) introduction in 2001 across 18 states [15]. In a post-hoc analysis of a randomized, placebo-controlled trial of 9-valent PCV (PCV9) in South Africa, PCV9 reduced pneumonia associated with any identified viruses, including RSV, influenza A, parainfluenza virus 1-3, and adenovirus, by 31%. For influenza A virus, only a 45% reduction was observed; however, the reduction in LRTIs cases associated with RSV only was not significant (22%; 95% confidence interval [CI] −3 to 41%) [14]. Hospitalization data during 1996-2012 in Western Australia did not show an impact of PCV on RSV infection, particularly, in Aboriginal infants [16]. In contrast a population-based birth cohort in Western Australia during 2000-2012 showed a 21-30% reduction in RSV hospitalization [17]. All these studies, mainly in high-income countries, suggest there may be a positive additional effect of PCV on RSV and influenza viral infections in young children; however, it is not clear whether this effect will also be observed inxbrk LMICs.

Mongolia has a very high burden of respiratory infections, with children being at the greatest risk [18]. The Government of Mongolia decided to introduce the 13-valent PCV (PCV13) (Prevenar^TM^, Pfizer) in a phased manner starting in Ulaanbaatar (UB), the capital city of Mongolia, from June 2016. A PCV Impact Evaluation Study was built on an existing World Health Organization–supported hospital-based lower respiratory tract infection (LRTI) surveillance system to document the impact of PCV13 on all-cause hospitalized LRTIs in children aged 2-59 months in four participating districts [19]. This PCV Impact Evaluation Study, supported by the Gavi Alliance, commenced in April 2015 and finished in June 2021. Using a nested study, we aimed to assess the impact of PCV13 on the incidence of severe, very severe LRTIs, and radiologically confirmed pneumonia associated with RSV and/or influenza in children under 2 years of age.

## Methods

### Study setting

This study used demographic and clinical data from the PCV Impact Evaluation Study [19]. PCV13 was introduced in a phased manner in the four study districts in UB. PCV13 was first introduced into two districts (Songinokhairkhan [SK] and Sukhbaatar [SB]) in June 2016, including a catch-up campaign for children aged up to 24 months (phase I). This was followed by the introduction of PCV13 into Bayanzurkh (BZ) district in July 2017, also with catch-up (phase II). The introduction into the fourth district (Chingeltei) and the remaining districts in UB occurred in March 2018 (phase III). The nationwide rollout was started in April 2019. The PCV13 schedule used in Mongolia is a 2 + 1 schedule (2, 4, and 9 months). A schematic of the study timeline is shown in Supplementary Figure 1.

During the COVID-19 pandemic, a countrywide lockdown was implemented in April 2020 [20]. Owing to the COVID testing burden in the main laboratory in UB, the collection and processing of nasopharyngeal swabs were halted twice: December 2020 to February 2021 and April to May 2021. Because of these disruptions, the PCV impact analysis was limited to the period April 2015 to March 2020.

### Study population

This nested study included children aged 2-23 months. All children meeting the following case definitions were eligible for this study and for RSV and influenza testing:

i. Severe LRTI: Acute illness associated with cough or breathing difficulty and fast breathing (respiratory rate >50) and arterial oxygen saturation (SaO_2_) less than 93% [21].
ii. Very severe LRTI: Acute illness associated with cough or breathing difficulty and fast breathing (respiratory rate >50) and (SaO_2_ less than 90%) or altered consciousness [21].
iii. Radiological pneumonia: Defined by World Health Organization criteria [22].

However, not all eligible children had nasopharyngeal swabs available owing to their delay in enrollment or refusal of nasopharyngeal swab collection (Supplementary Table 1; Supplementary Figure 2).

### Data and samples collection

Data, samples collections, and RSV and influenza virus testing were previously described [19,23].

### Descriptive analysis

Clinical and demographic characteristics and the risk factors potentially associated with severe LRTIs, as well as the severity of LRTIs, were described for the whole study population and stratified by PCV introduction period. Continuous variables were summarized with median and interquartile ranges. Categorical variables were summarized with frequency counts and percentages. Comparisons were analyzed using the Mann–Whitney U test for continuous variables and the Fisher exact test for categorical variables.

### Primary and secondary outcomes of the impact of PCV 13 analysis

Our primary outcome was the impact of PCV13 on the incidence rates of severe LRTIs associated with RSV. The secondary outcomes were the impact of PCV13 on the incidence rates of:

i. Very severe LRTI associated with RSV,
ii. Radiological pneumonia associated with RSV,
iii. Severe LRTI associated with influenza,
iv. Very severe LRTI associated with influenza, and
v. Radiological pneumonia associated with influenza.

LRTIs were considered associated with RSV or influenza if these viruses were detected on polymerase chain reaction testings.

For the main analysis, the post-PCV period was defined from the PCV13 introduction time point. Based on the defined post-PCV period, we calculated the incidence rates during the pre- and post-PCV13 periods and incidence rate ratios (IRRs) comparing the pre- and post-PCV13 periods for the primary and secondary outcomes for all four districts and stratified by district. Because RSV mainly affects children less than 6 months of age [1], IRRs of LRTIs associated with either RSV or influenza infection were also conducted in three separate age groups (under 6 months, 6 months to 1 year, and older than 1 year).

Crude incidence rates for primary and secondary outcomes were calculated by dividing the numbers of cases of each outcome by the annual population in each district provided by the Mongolian Ministry of Health and expressed per 1000 population. Confidence intervals (CIs) for incidence estimates were calculated using a Poisson distribution.

Unadjusted IRRs (uIRRs) and their 95% CIs associated with each outcome in each district were analyzed using monthly case numbers as the outcomes in negative binomial regression models. The natural logarithm of the population denominators was included as an offset and a dummy variable was included to represent the PCV13 introduction period in these models (district specific, unadjusted model). To calculate the adjusted IRRs (aIRRs) for each outcome for individual districts, we also included the chronological period as a continuous variable to adjust for underlying secular trends and calendar month as categorical variable to adjust for seasonality in the negative binomial regression models [24] (district specific, adjusted model).

For overall estimates for all four districts together, mixed-effects negative binomial regression models were used with a fixed effect for the PCV13 introduction variable and a random effect for the district to calculate the uIRR (overall unadjusted model). To calculate the aIRRs for all four districts, mixed-effects negative binomial regression models had additional fixed-effect variables for chronological period as a continuous variable to adjust for the underlying secular trends and calendar month as categorical variable to adjust for seasonality [24] (overall adjusted model). The coefficients of all the previously mentioned models were exponentiated to obtain the IRRs for the PCV13 effect.

### Sensitivity analyses

Two additional sensitivity analyses were performed to assess the robustness of our findings. First, we assumed there was a 1-year delayed PCV13 impact (e.g. 1 year lag period) from PCV introduction, including 1 year transition (the primary analyses assumed no lag in impact).

Second, we also checked the PCV13 impact on the incidence of severe LRTIs among cases testing negative for RSV and influenza. It is plausible to assume that this virus negative, severe group would be more impacted by PCV13 because the patients in this group were more likely to have *S. pneumoniae*.

All statistical analysis was implemented using Stata version 16.0 (StataCorp LP, College Station, TX, USA) and R version 4.3.0 (R Foundation for Statistical Computing, Vienna). The 95% CIs were given for each incidence rate and incidence rate ratio. All statistical tests were conducted at the two-tailed 5% significance level.

## Results

### Clinical and demographic characteristics of patients pre- and post-PCV 13 introduction

The characteristics of study participants, potential LRTI risk factors, and LRTI severity, as well as the prevalence of RSV/influenza infection before and after PCV13 introduction in all districts and in each individual district are presented in Table 1 and Supplementary Table 2, respectively.

**Table 1:** Clinical and demographic characteristics of subjects in the study pre- and post- PCV introduction in all districts (April 2015-March 2020).

Category	All districts (N = 5577)	
	Pre- PCV (N = 2347)	Post-PCV (N = 3230)
Sex (male) n, %	1316 (56)	1882 (56)
Under 6 months of age	521 (22)	876 (27)
Number of siblings <5 years	771(33)	1093(34)
Crowding (>3 people per room)	1634 (70)	2342 (73)
Smokers in the home	253 (11)	331 (10)
Smokey Fuel for cooking	765 (33)	1160 (36)
Ger house	960 (41)	1224 (38)
Household income at/below minimum poverty level	859 (37)	1357 (42)
Household member treated for tuberculosis	35 (2)	39 (1)
Asthma	215 (9)	210 (7)
Malnutrition	217 (9)	241 (7)
History of measles infection	104 (4)	12 (0)
Length of hospital stay (>7 days)	1207 (51)	1528 (47)
Previous admission for pneumonia	795 (34)	977 (30)
Antibiotic given 48 hours before hospital	1228 (52)	1610 (50)
Antibiotic given in hospital	2179 (93)	2901 (90)
O_2_ supplementation	968 (41)	1354 (42)
Hypoxia (O_2_ saturation <90%)	844 (36)	1117 (35)
Severe LRTIs	1470 (63)	2009 (62)
Very severe LRTIs	659 (28)	807 (25)
Xray (+)	498 (21)	721 (22)
Death	8 (0)	8 (0)
Respiratory syncytial virus (+)	730 (31)	1378 (43)
Influenza (+)	158 (7)	229 (7)

LRTI, lower respiratory tract infections; PCV, pneumococcal conjugate vaccine.

No significant differences in clinical and demographic characteristics of patients in the overall district data pre- and post-PCV introduction were noted (Table 1); however, some significant differences were noted in individual districts, SK and BZ, pre- and post-PCV introduction (Supplementary Table 2). During the post-PCV13 introduction period, the risks potentially associated with severe LRTIs, such as smoker in the household, ger house type (housing type), asthma, malnutrition, and history of previous admission for pneumonia, as well as the severity of LRTIs reflected through the duration of hospitalization, oxygen supplementation, and the prescription of antibiotics were significantly reduced in SK district. In contrast, in BZ, during the post-PCV13 introduction period, the risks potentially associated with severe LRTIs, such as young children under 6 months of age, living in crowded household, and low household income were significantly increased (Supplementary Table 2).

Supplementary Figure 3 shows the incidences of RSV and influenza cases in each district during April 2015-March 2020. Except for the 2016 season, in all districts, RSV and influenza peaks coincided with the winter months, started in November and finished in March. However, during the 2016 season, the virus peaks were delayed, started in January and finished in May.

### Impact of PCV 13 on the incidence outcomes associated with RSV or with influenza virus

**Figure 1:**
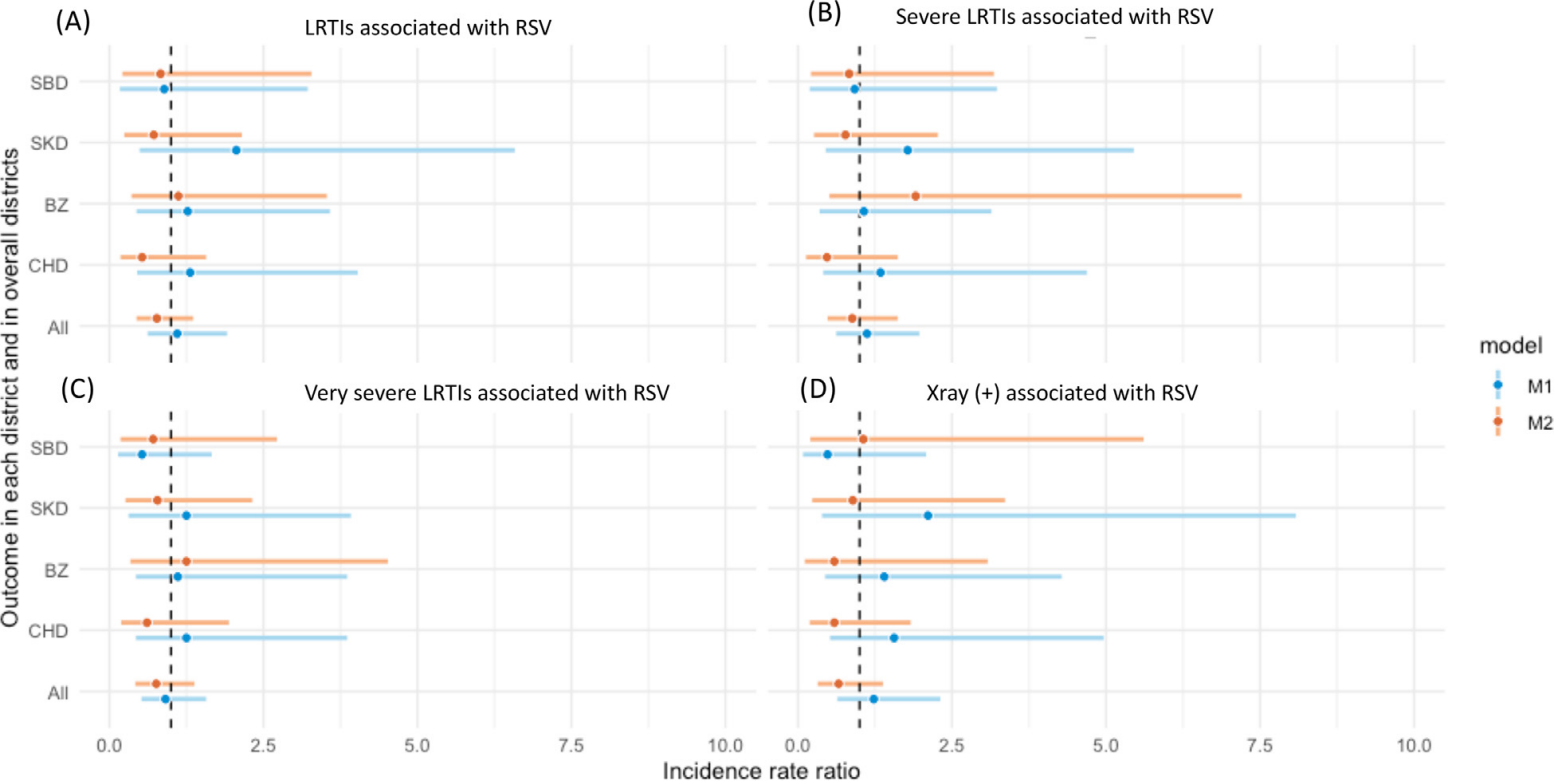
IRRs of outcomes associated with RSV infection: LRTIs (a), severe LRTIs (b), very severe LRTIs (c) and X-ray confirmed pneumonia (d) comparing pre- and post- PCV13 introduction. Comparing pre- and post- PCV13 introduction. *Model 1 (M1- unadjusted for seasonality):* - For each district SBD, SKD, BZ and CHD (district specific, unadjusted model): crude IRRs with each outcome were calculated using monthly count data and negative binomial regression models. The natural logarithm of the population denominators was included as an offset and a dummy variable was included to represent PCV13 introduction period in these models*.* - For overall estimates of all four districts (all overall districts, unadjusted model): unadjusted IRRs with each outcome were calculated by using mixed effects negative binomial regression models with a fixed effect for PCV13 introduction variable and a random effect for district. *Model 2 (M2- adjusted for seasonality)* - For each district SBD, SKD, BZ and CHD (district specific, adjusted model for seasonality): adjusted IRRs with each outcome were calculated for individual district using a negative binomial model that included chronological time period as a continuous variable to adjust for underlying secular trends and calendar month as categorical variable to adjust for seasonality. - For overall estimates of all four districts (All overall districts, adjusted model): adjusted IRRs with each outcome were calculated by using mixed effects negative binomial regression models with a fixed effect for PCV13 introduction variable, a random effect for district and additional fixed effect variables for chronological time period as a continuous variable to adjust for underlying secular trends and calendar month as categorical variable to adjust for seasonality. BZ, Bayanzurkh; CHD, Chingeltei; IRR, incidence rate ratio; LRTI, lower respiratory tract infections; PCV, pneumococcal conjugate vaccine; RSV, respiratory syncytial virus; SBD,Sukhbaatar; SKD, Songinokhairkhan.

**Figure 2:**
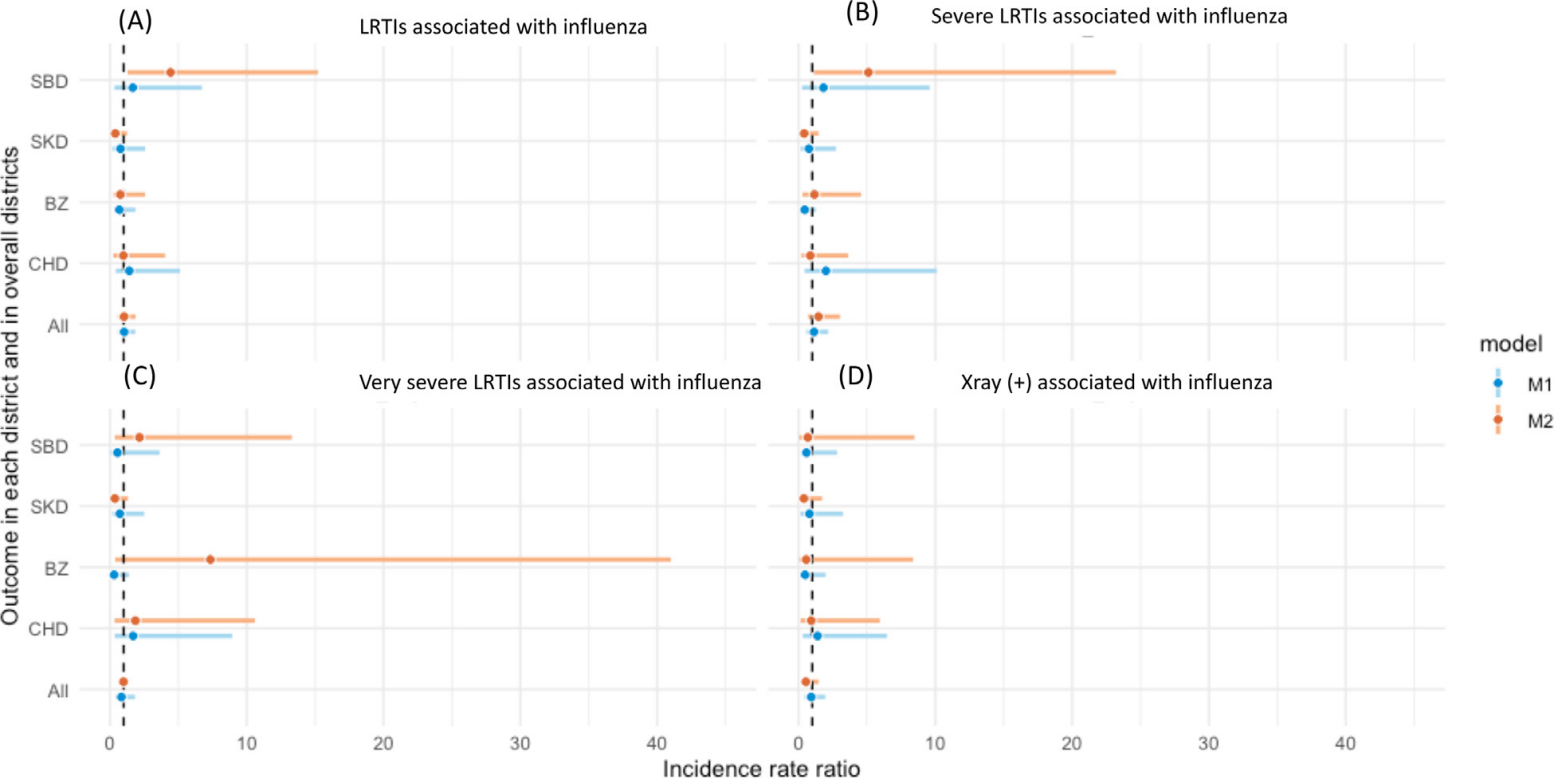
IRRs of outcomes associated with influenza infection: LRTIs (a), severe LRTIs (b), very severe LRTIs (c) and X-ray confirmed pneumonia (d) comparing pre- and post- PCV13 introduction. *Model 1 (M1-unadjusted for seasonality):* - For each district SBD, SKD, BZ and CHD (district specific, unadjusted model): crude IRRs with each outcome were calculated using monthly count data and negative binomial regression models. The natural logarithm of the population denominators was included as an offset and a dummy variable was included to represent PCV13 introduction period in these models*.* - For overall estimates of all four districts (All overall districts, unadjusted model): unadjusted IRRs with each outcome were calculated by using mixed effects negative binomial regression models with a fixed effect for PCV13 introduction variable and a random effect for district. *Model 2 (M2- adjusted for seasonality)* - For each district SBD, SKD, BZ and CHD (district specific, adjusted model for seasonality): adjusted IRRs with each outcome were calculated for individual district using a negative binomial model that included chronological time period as a continuous variable to adjust for underlying secular trends and calendar month as categorical variable to adjust for seasonality. - For overall estimates of all four districts (all overall districts, adjusted model): adjusted IRRs with each outcome were calculated by using mixed effects negative binomial regression models with a fixed effect for PCV13 introduction variable, a random effect for district and additional fixed effect variables for chronological time period as a continuous variable to adjust for underlying secular trends and calendar month as categorical variable to adjust for seasonality. BZ, Bayanzurkh; CHD, Chingeltei; IRR, incidence rate ratio; LRTI, lower respiratory tract infections; PCV, pneumococcal conjugate vaccine; RSV, respiratory syncytial virus; SBD, Sukhbaataar; SKD, Songinokhairkhan.

Figure 1, Figure 2 show the uIRR and aIRR results for each outcome in individual districts and in all districts combined. Supplementary Table 3 provides the details of these results. Non-significant impact was observed in unadjusted results. When adjusted for seasonality, we observed a non-significant decreased trend in all outcomes associated with RSV infection in the post-PCV13 introduction period in the three districts: SK, SB, and CHD, as well overall (Figure 1). One district, BZ district, demonstrated an increased trend in LRTIs, severe LRTIs, and very severe LRTIs associated with RSV but a decreased trend in X-ray–confirmed pneumonia (Figure 1), but none were statistically significant.

The trends of changes in outcomes associated with influenza were inconsistent across the districts and outcomes and remained non-significant. Influenza outcomes had uIRRs and aIRRs with much larger CIs than those of RSV outcomes (Figure 2).

The analyses for children under 6 months, for those 6 months to 1 year, and for those older than 1 year revealed non-significant changes associated with RSV or influenza infection outcomes across the three age groups in any individual district, as well as all districts (Table 2).

**Table 2:** The impact of PCV13 vaccine on incidence rate ratios of LRTIs associated with respiratory syncytial virus or with viral infections by district in the pre- and post-PCV13 period, April 2015-March 2020 in children under 6 months and in children older than 1 year.

Outcomes	District	Children under 6 months		Children 6-month -1 year		Children older than 1 year	
		Incidence rate ratio	Incidence rate ratio	Incidence rate ratio	Incidence rate ratio	Incidence rate ratio	Incidence rate ratio
		(unadjusted)	(adjusted)	(unadjusted)	(adjusted)	(unadjusted)	(adjusted)
LRTIs associated with respiratory syncytial virus	All	1.2 (0.7-2.2)	0.8 (0.4-1.4)	1.1 (0.5-2.0)	0.7 (0.4-1.1)	1.0 (0.6-1.8)	0.9 (0.5-1.5)
	CHD	1.5 (0.5-5.0)	0.5 (0.2-1.4)	1.5 (0.4-5.8)	0.4 (0.1-1.3)	1.1 (0.4-3.1)	0.6 (0.2-1.8)
	BZ	1.5 (0.5-4.7)	1.1 (0.3-4.1)	1.0 (0.3-3.6)	1.6 (0.3-8.2)	1.3 (0.5-3.1)	1.5 (0.5-4.4)
	SKD	2.1 (0.5-7.1)	1.0 (0.3-3.2)	1.8 (0.4-6.8)	1.0 (0.3-2.9)	2.2 (0.5-7.4)	0.7 (0.2-2.4)
	SBD	1.0 (0.2-3.8)	0.9 (0.2-3.8)	0.9 (0.2-0.4)	0.9 (0.2-4.0)	0.8 (0.2-3.0)	0.8 (0.2-3.3)
LRTIs associated with viral infections	All	1.3 (0.7-2.3)	0.8 (0.5-1.5)	1.1 (0.6-2.0)	0.8 (0.7-0.8)	1.0 (0.6-1.6)	0.9 (0.5-1.4)
	CHD	1.5 (0.5-5.2)	0.5 (0.2-1.4)	1.5 (0.4-5.7)	0.6 (0.2-1.7)	1.1 (0.4-2.8)	0.7 (0.2-2.0)
	BZ	1.6 (0.5-4.8)	1.2 (0.3-4.5)	1.0 (0.3-2.8)	1.4 (0.4-5.0)	1.1 (0.5-2.4)	1.3 (0.6-3.0)
	SKD	2.1 (0.6 -6.7)	1.1 (0.4-3.2)	1.8 (0.4-6.2)	1.1 (0.4-2.7)	1.3 (0.4-3.7)	0.6 (0.3-1.4)
	SBD	1.1 (0.2-4.1)	0.9 (0.2-3.8)	0.9 (0.2-3.1)	1.4 (0.4-4.7)	0.9 (0.2-2.8)	1.2 (0.3-4.0)

BZ, Bayanzurkh; CHD, Chingeltei; LRTI, lower respiratory tract infections; PCV, pneumococcal conjugate vaccine; SBD, Subkhbaatar; SKD, Songinokhairkhan.

### Sensitivity analyses

Although still non-significant, when we analyzed the data with a 1-year lag period, we observed clearer trend changes toward a reduction of different outcomes associated with RSV in all districts combined (Supplementary Figure 4 and Supplementary Table 4).

For the outcomes associated with influenza infection, with the 1-year lag scenario, no consistent results were observed (Supplementary Figure 5 and Supplementary Table 4). Significant increases by 1.11-1.65 times in very severe LRTIs and X-ray–confirmed pneumonia associated with influenza were observed in all districts combined (Supplementary Table 4).

When we restricted our analysis to the viral negative LRTI group, we observed a significant reduction in adjusted models in the main analysis (aIRR 0.79, 95% CI 0.66-0.95) and in the 1-year lag analysis (aIRR 0.68, 95% CI 0.52-0.90) for all four districts combined (Supplementary Figure 6 and Supplementary Table 4).

## Discussion

In this study, we evaluated the impact of PCV13 introduction on the incidence rates of hospitalized LRTIs associated with RSV and influenza infections in children under 2 years of age. This included a series of sensitivity analyses to examine the robustness of results to different modeling decisions.

Our study could not replicate the significant impact of PCV in reducing the incidence of LRTIs associated with RSV or influenza reported in previous ecological studies [15], [16], [17]. However, we still observed trends consistent with reductions in outcomes associated with RSV infection when adjusted for seasonality and time secular trends, particularly, in all four districts combined. Introducing a 1-year lag effect showed clearer reduction trends in RSV outcomes and a significant reduction in LRTIs associated with viral negative LRTI cases. The finding in viral negative cases confirmed our current models can capture the direct effect of PCV13 on the outcome likely associated with *S. pneumoniae*. The magnitude of PCV13 impact on this outcome aligned with those observed in the main surveillance data [25].

Because RSV and influenza had a strong seasonal pattern and their incidences varied strongly between years, with the adjustment for seasonality by month, a shifted trend of changes in all outcomes was observed (Figures 1 and 2). Adequate control for seasonality is crucial and complex because seasonal variations can mask the direct impact of PCV. Using data from Israel, Weinberger *et al.* [26] demonstrated that when controlling for seasonality a clearer significant impact of PCV on the incidence of a radiologically confirmed alveolar pneumonia (RCAP) and in young children in the first 2 years after vaccine introduction was observed.

Of note, RSV seasons vary in severity (e.g. number of cases) owing to a range of factors, including variations in temperature [27] and pollution levels [28]. Although these factors were not included in the current analysis and our current approach to control the seasonality factor was not specific to a pathogen, it still provided a sufficient control measures of these factors, which are also strongly associated with seasonality.

There are several factors that challenged our investigation on the impact of PCV13 on RSV or influenza outcomes in our study context. First, our database was based on hospitalization cases, which are less sensitive to detect PCV impact, as reported in previous studies. Weinberger *et al.* observed a strong relationship between RSV incidence and RCAP across the entire study period but when RSV was removed from their model, this had little or no effect on the estimates of vaccine impact in any of the age groups [26]. Their hospitalized RCAP data could not rule out PCV impact on RSV infection nor detect the reduction trend in the RSV data. Similarly, hospitalization data during 1996-2012 in Western Australia failed to show a clear reduction trend on RSV infection [16], whereas the population-based data from the same state during 2000-2012 demonstrated a significant positive impact of PCV on RSV infection [17]. In contrast, the hospital-based surveillance of community-acquired pneumonia in France showed an increase trend in all virus detection in community-acquired pneumonia cases (1.4%, 95% CI 0.3-2.6%) per month after PCV introduction [29].

Inconsistent findings derived from hospitalization databases may be explained by the influences of changes in health care delivery before and after PCV introduction. These changes can be verified by exploring the association between hospitalization rates and health care access and quality [30]. With very little inpatient care of children in the private sector in Mongolia, almost all sick children have access to the participating public district/referral hospitals in our study during the pre- and post-PCV introduction periods. The health care quality does not vary much between public sector facilities. However, it is possible that the hospitalization threshold varied, especially during the high RSV season and because of the reductions of LRTIs associated with *S. pneumoniae* as the direct impact of PCV13.

Second, we observed variability in the proportion of young children aged less than 6 months and some risk factors potentially associated with increased severity of LRTIs between the pre- and post-PCV introduction periods across the four districts (Supplementary Table 2). These differences potentially contributed to the variability of PCV13 impact across districts. A possible approach to test this hypothesis is to check the impact on stratified groups with and without the risk factors; however, the RSV positive sample size did not have enough power to explore this and we do not have specific denominators for each risk factor stratification [31]. Our analysis for the under 6 months and older than 1 year old groups did not find differences. We used the mixed-effect model to address the variability across the four districts. A higher number of RSV cases in the overall district data set with greater power resulted in more consistent RSV trends observed in the mixed-effect models than the trends in individual district (Figure 1).

Third, our analyses were subject to two major confounders that could have influenced our findings. The largest national measles outbreak in Mongolia over the past 20 years occurred in the pre-PCV introduction period (2015-2016) [32] and the peak of the COVID-19 pandemic occurred in the post-PCV introduction period (2020-2021) [20]. We observed a correlation between measles deaths and the peak of RSV detection and a dramatic increase in all (severe) LRTIs associated with RSV but not with influenza A or B in the year after the measles virus outbreak [32]. However, only 116 study participants with a history of measles infection during this outbreak were identified from archived medical documents (Do *et al.* manuscript in preparation). Of note, there were 23,464 and 30,273 measles cases in 2015 and in 2016, respectively. UB city had more than 30,000 cases [33]. Hence, we were not confident that we had sufficient measles data to examine its potentially confounding role in the impact of PCV analysis.

The COVID-19 pandemic disrupted our data collection and the analysis plan. Because we had only 1 year for pre-PCV13 introduction, the phased introduction design allowed additional data pre-PCV13 introduction in phase II and phase III districts. However, because of the disruption of the COVID-19 pandemic, our post-PCV13 introduction data were limited to March 2020, missing 14 months post-PCV13 data (April 2020-June 2021). This disruption crucially limited our evaluation of PCV13 impact. In addition, COVID-19 pandemic has been reported to significantly impact RSV and influenza epidemiology [34]. It is still unclear how RSV and influenza epidemiology evolves in the post-COVID era; hence, this uncertainty also challenges the evaluation of PCV13 impact on RSV and influenza infection when using surveillance data.

Weinberger *et al.* [15] found that the impact of PCV7 on RSV hospitalizations in children aged 3-11 months only started to be observed from the second- and third-year post-PCV7 introduction. We conducted an analysis using a 1-year lag period which included the year after vaccine introduction (considered as a transition year). We investigated whether the magnitude of the vaccine effects could be better demonstrated. Although there was a greater reduction trend, it remained non-significant. The short post-PCV13 period because of the disruption of COVID pandemic also limited the 1-year lag analysis.

Fourth, the study design has some limitations. Our study was designed in collaboration with the Government of Mongolia. Initially, our study was designed as a step wedge with over 20 areas; however, this approach was not logistically possible and replaced by a three-step introduction. The reduced number of participants of each step because of the COVID-19 pandemic disruption was an unpredictable element of the analysis plan. In addition, Mongolian populations tend to be mobile and it is not unusual for people living in traditional ger dwellings to move around the city [35], creating uncertainty in the denominators. Moreover, although the district hospitals of our study served 70% of the UB population, our study population only focused on severe cases in children under 2 years of age; therefore, the study findings can only be applied for that population. Missing NP swabs during the first year before PCV introduction were also an important factor affecting the evaluation of PCV impact. Conducting an imputation analysis is a challenge because the missing swabs were not at random.

## Conclusion

In conclusion, the impact of PCV13 on the outcomes associated with RSV infection varied across districts and appeared more prominent in the second year of use. The impact of PCV13 on the outcomes associated with influenza virus infection were inconclusive, likely because of the low number of influenza cases detected. Besides a high incidence of severe RSV hospitalization, from our data, Mongolia also has a high prevalence of potential social and epidemiological risk factors associated with severe RSV infections. These factors may influence the PCV13 impact in our data and highlights the importance of including those factors in future evaluations of RSV prevention in Mongolia, as well as in any LMICs.

## Declarations of competing interest

The authors have no competing interests to declare.

## Appendix Supplementary materials

Image, application 1
