# Supplementary material for: Impact of pneumococcal conjugate vaccine 13 introduction on severe lower respiratory tract infections associated with respiratory syncytial virus or influenza virus in hospitalized children in Ulaanbaatar, Mongolia
Source: IJID Reg. 2024 Mar 19;11:100357. doi: 10.1016/j.ijregi.2024.100357 (PMC10992709; doi:10.1016/j.ijregi.2024.100357)
Supplement: Supplementary file 1 [file mmc1.zip › LAHD_Supp.Figures_IJID- final_v2.docx]

**Supp. Figure 1** **- Schematic of the study periods. Green cells represent time periods after PCV13 introduction.**

*
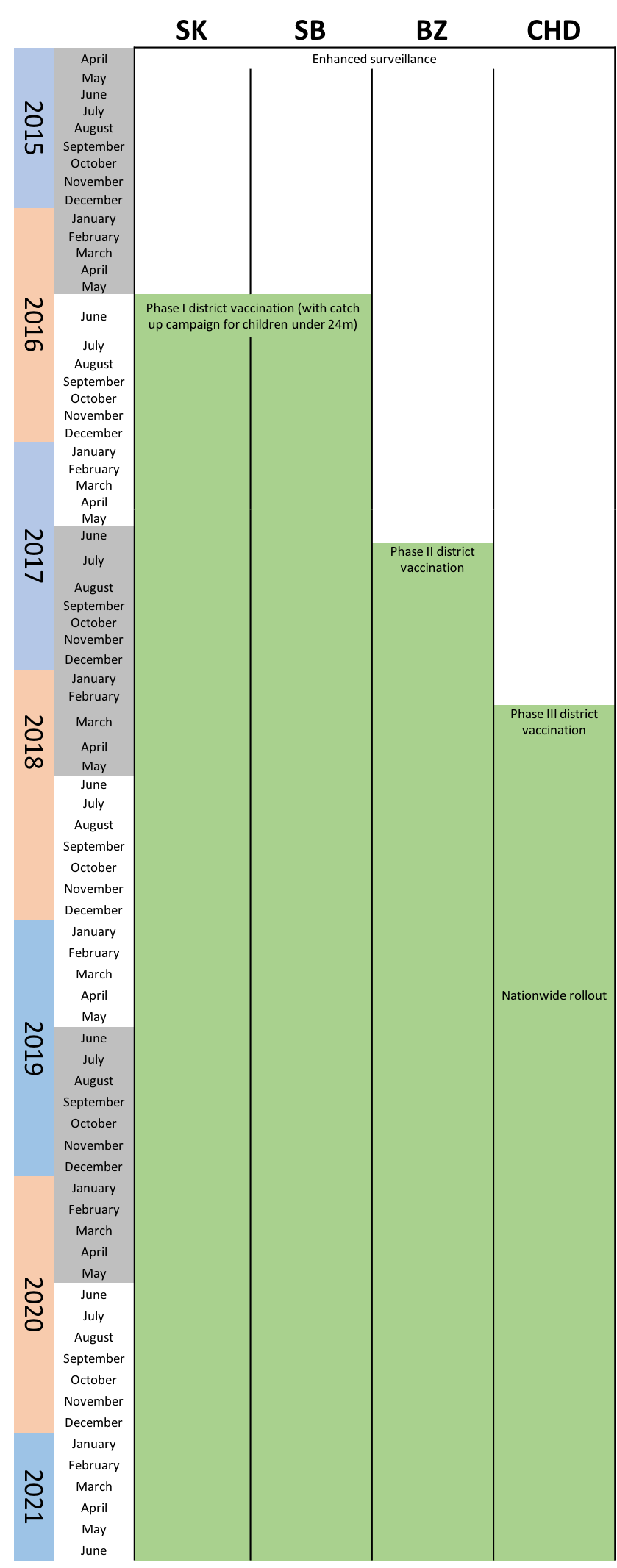
*

*(SK:Songinokhairkhan , SB: Sukhbataar, BZ:Bayanzurkh CHD:Chingeltei )*

**Supp. Figure 2- Incidence rate of eligible cases for RSV and influenza testing and the actual tested cases in each district (April 2015-March 2020).**

***
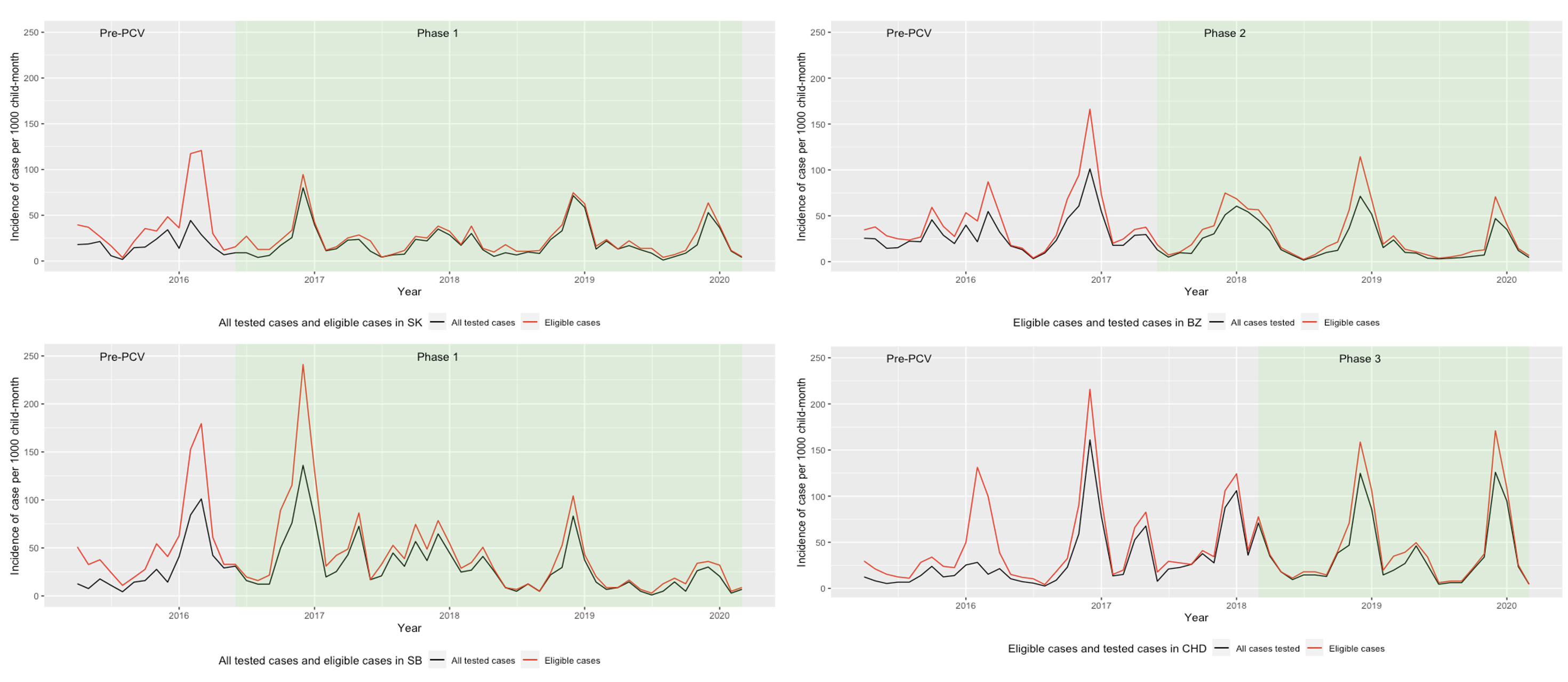
***

**Supp. Figure 3 – Incidences of RSV and influenza cases in each district, across study time used for impact evaluation (April 2015-March 2020). Green cells represent time periods after PCV13 introduction.**

***(SK:Songinokhairkhan , SB: Sukhbataar, BZ:Bayanzurkh CHD:Chingeltei )***

***
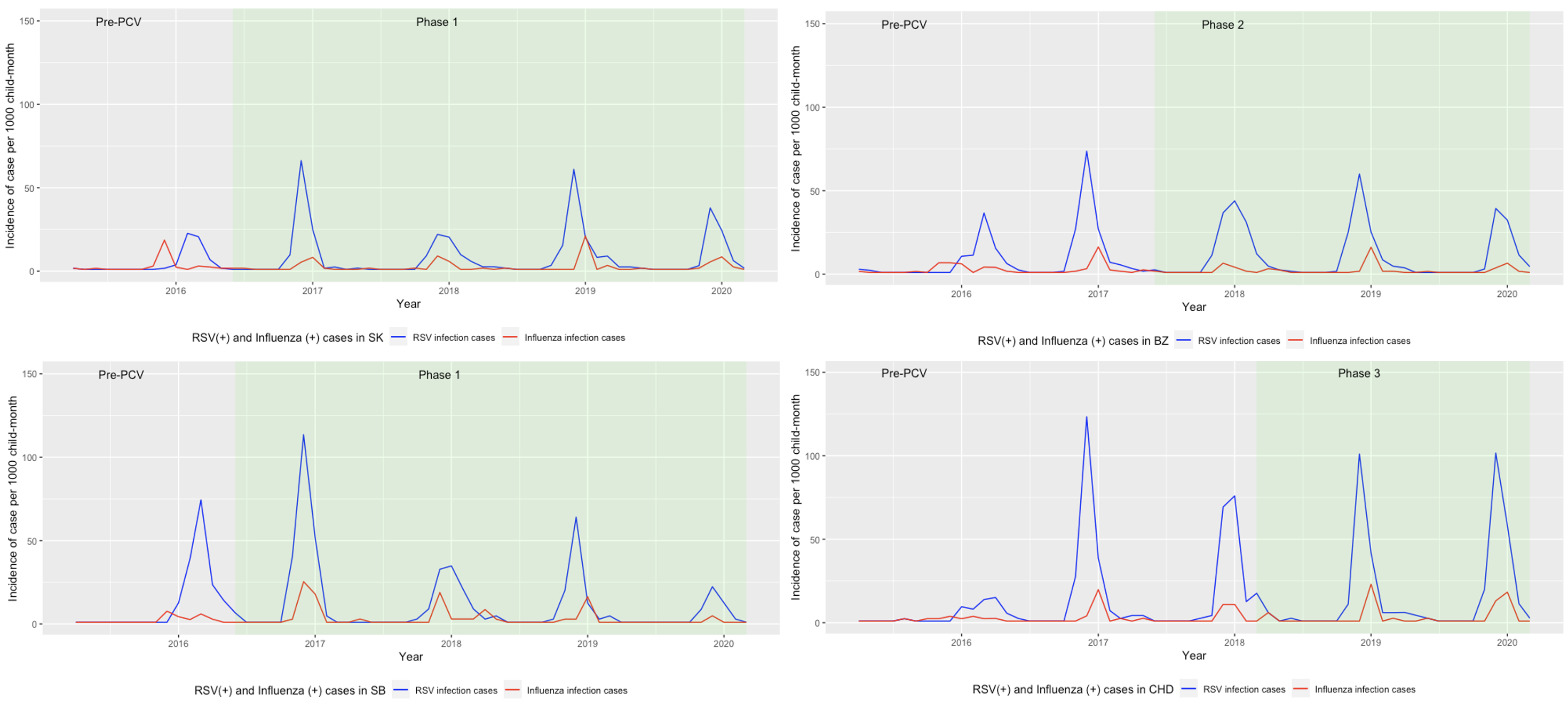
***

***Supp. Figure 4-Sensitivity analysis.***

Incidence rate ratios (IRRs) of incidences of outcomes associated with RSV infection in main analysis (M1 and M2) and one-year lag analysis (M1a and M2a): LRTIs (A), severe LRTIs (B), very severe LRTIs (C) and Xray confirmed pneumonia (D) comparing pre- and post- PCV13 introduction.

*Model 1 (M1 – unadjusted for seasonality):*

- For each district SBD, SKD, BZ and CHD (district specific, unadjusted model):  *crude IRRs with each outcome were calculated* using monthly count data and negative binomial regression models. The natural logarithm of the population denominators was included as an offset and a dummy variable was included to represent PCV13 introduction period in these models*.*
- For overall estimates of all four districts (All overall districts, unadjusted model): unadjusted IRRs with each outcome were calculated by using mixed effects negative binomial regression models with a fixed effect for PCV13 introduction variable and a random effect for district.

*Model 1a (M1a – unadjusted for seasonality with one-year lag):* the IRRs were calculated using the same approach in M1 for individual district estimate and for the overall estimates of all four district, but with the assumption that the start of the post-PCV period was one-year delayed from the PCV13 introduction time point.

*Model 2 (M2- adjusted for seasonality)*

- For each district SBD, SKD, BZ and CHD (district specific, adjusted model for seasonality): adjusted IRRs (aIRR) with each outcome *were calculated for individual district using a negative binomial model* that included chronological time period as a continuous variable to adjust for underlying secular trends and calendar month as categorical variable to adjust for seasonality.
- For overall estimates of all four districts (All overall districts, adjusted model): *adjusted IRRs with each outcome were calculated* by using mixed effects negative binomial regression models with a fixed effect for PCV13 introduction variable, a random effect for district and additional fixed effect variables for chronological time period as a continuous variable to adjust for underlying secular trends and calendar month as categorical variable to adjust for seasonality.

*Model 2a (M2a – adjusted for seasonality with one-year lag):* the aIRRs were calculated using the same approach in M2 for individual district estimate and for the overall estimates of all four district, but with the assumption that the start of the post-PCV period was one year delayed from the PCV13 introduction time point*.*

*
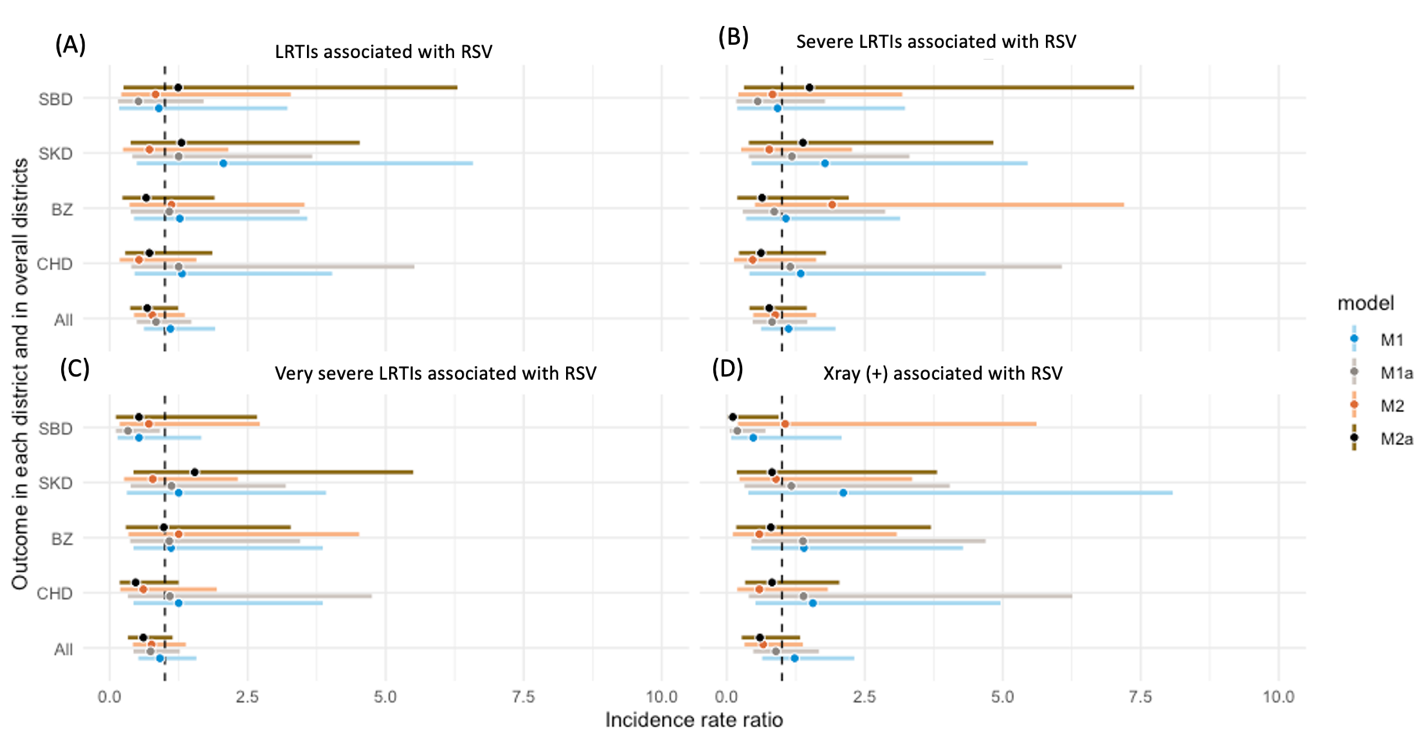
*

**Supp.Figure 5- Incidence rate ratios (IRRs) of incidences of outcomes associated with influenza infection in primary (M1 and M2) and in secondary scenarios (M1a, and M2a): LRTIs (A), severe LRTIs (B), very severe LRTIs (C) and Xray confirmed pneumonia (D) comparing pre- and post- PCV13 introduction.**

*Model 1 (M1 – unadjusted for seasonality):*

- For each district SBD, SKD, BZ and CHD (district specific, unadjusted model):  *crude IRRs with each outcome were calculated* using monthly count data and negative binomial regression models. The natural logarithm of the population denominators was included as an offset and a dummy variable was included to represent PCV13 introduction period in these models*.*
- For overall estimates of all four districts (All overall districts, unadjusted model): unadjusted IRRs with each outcome were calculated by using mixed effects negative binomial regression models with a fixed effect for PCV13 introduction variable and a random effect for district.

*Model 1a (M1a – unadjusted for seasonality with one-year lag):* the IRRs were calculated using the same approach in M1 for individual district estimate and for the overall estimates of all four district, but with the assumption that the start of the post-PCV period was one-year delayed from the PCV13 introduction time point.

*Model 2 (M2- adjusted for seasonality)*

- For each district SBD, SKD, BZ and CHD (district specific, adjusted model for seasonality): adjusted IRRs (aIRR) with each outcome *were calculated for individual district using a negative binomial model* that included chronological time period as a continuous variable to adjust for underlying secular trends and calendar month as categorical variable to adjust for seasonality
- For overall estimates of all four districts (All overall districts, adjusted model): *adjusted IRRs with each outcome were calculated* by using mixed effects negative binomial regression models with a fixed effect for PCV13 introduction variable, a random effect for district and additional fixed effect variables for chronological time period as a continuous variable to adjust for underlying secular trends and calendar month as categorical variable to adjust for seasonality

*Model 2a (M2a – adjusted for seasonality with one-year lag):* the aIRRs were calculated using the same approach in M2 for individual district estimate and for the overall estimates of all four district, but with the assumption that the start of the post-PCV period was one year delayed from the PCV13 introduction time point*.*


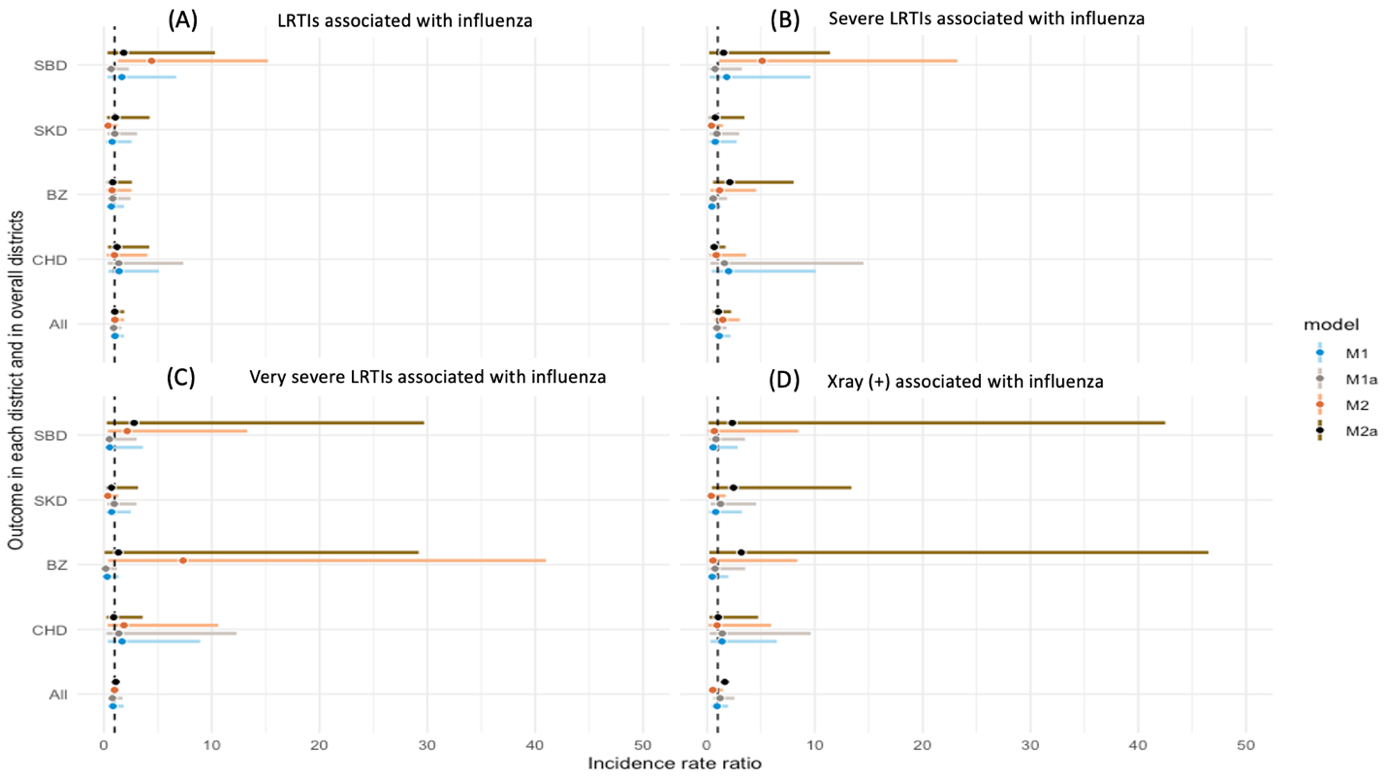


**Supp. Figure 6- Incidence rate ratios (IRRs) of incidences of outcomes associated with negative viral infection in primary (M1 and M2) and in secondary scenarios (M1a and M2a) comparing pre- and post- PCV13 introduction.**

*Model 1 (M1 – unadjusted for seasonality):*

- For each district SBD, SKD, BZ and CHD (district specific, unadjusted model):  *crude IRRs with each outcome were calculated* using monthly count data and negative binomial regression models. The natural logarithm of the population denominators was included as an offset and a dummy variable was included to represent PCV13 introduction period in these models*.*
- For overall estimates of all four districts (All overall districts, unadjusted model): unadjusted IRRs with each outcome were calculated by using mixed effects negative binomial regression models with a fixed effect for PCV13 introduction variable and a random effect for district.

*Model 1a (M1a – unadjusted for seasonality with one-year lag):* the IRRs were calculated using the same approach in M1 for individual district estimate and for the overall estimates of all four district, but with the assumption that the start of the post-PCV period was one-year delayed from the PCV13 introduction time point.

*Model 2 (M2- adjusted for seasonality)*

- For each district SBD, SKD, BZ and CHD (district specific, adjusted model for seasonality): adjusted IRRs (aIRR) with each outcome *were calculated for individual district using a negative binomial model* that included chronological time period as a continuous variable to adjust for underlying secular trends and calendar month as categorical variable to adjust for seasonality
- For overall estimates of all four districts (All overall districts, adjusted model): *adjusted IRRs with each outcome were calculated* by using mixed effects negative binomial regression models with a fixed effect for PCV13 introduction variable, a random effect for district and additional fixed effect variables for chronological time period as a continuous variable to adjust for underlying secular trends and calendar month as categorical variable to adjust for seasonality

*Model 2a (M2a – adjusted for seasonality with one-year lag):* the aIRRs were calculated using the same approach in M2 for individual district estimate and for the overall estimates of all four district, but with the assumption that the start of the post-PCV period was one year delayed from the PCV13 introduction time point*.*

**
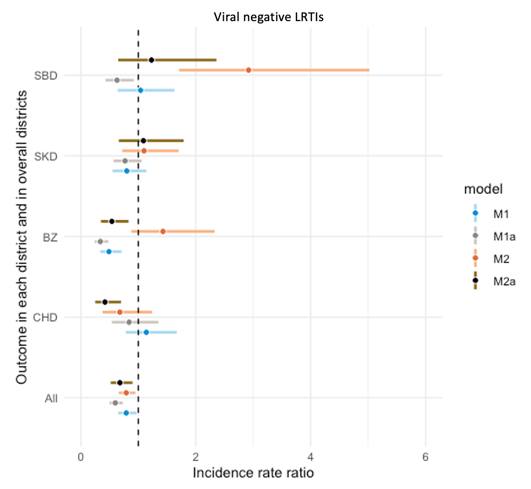
**
